# Application of machine learning models in predicting length of stay among healthcare workers in underserved communities in South Africa

**DOI:** 10.1186/s12960-018-0329-1

**Published:** 2018-12-13

**Authors:** Sangiwe Moyo, Tuan Nguyen Doan, Jessica Ann Yun, Ndumiso Tshuma

**Affiliations:** 1 Africa Health Placements, Rosebank, Johannesburg, South Africa; 2 Yale University, New Haven, CT United States of America; 3 The Best Health Solutions, 107 Louis Botha Avenue, Orange Grove, Norwood, P.O. Box 92666, Johannesburg, South Africa

**Keywords:** Machine learning, Artificial intelligence, Health workers, Modeling, Staff retention

## Abstract

**Background:**

Human resource planning in healthcare can employ machine learning to effectively predict length of stay of recruited health workers who are stationed in rural areas. While prior studies have identified a number of demographic factors related to general health practitioners’ decision to stay in public health practice, recruitment agencies have no validated methods to predict how long these health workers will commit to their placement. We aim to use machine learning methods to predict health professional’s length of practice in the rural public healthcare sector based on their demographic information.

**Methods:**

Recruitment and retention data from Africa Health Placements was used to develop machine-learning models to predict health workers’ length of practice. A cross-validation technique was used to validate the models, and to evaluate which model performs better, based on their respective aggregated error rates of prediction. Length of stay was categorized into four groups for classification (less than 1 year, less than 2 years, less than 3 years, and more than 3 years). R, a statistical computing language, was used to train three machine learning models and apply 10-fold cross validation techniques in order to attain evaluative statistics.

**Results:**

The three models attain almost identical results, with negligible difference in accuracy. The “best”-performing model (Multinomial logistic classifier) achieved a 47.34% [SD 1.63] classification accuracy while the decision tree model achieved an almost comparable 45.82% [SD 1.69]. The three models achieved an average AUC of approximately 0.66 suggesting sufficient predictive signal at the four categorical variables selected.

**Conclusions:**

Machine-learning models give us a demonstrably effective tool to predict the recruited health workers’ length of practice. These models can be adapted in future studies to incorporate other information beside demographic details such as information about placement location and income. Beyond the scope of predicting length of practice, this modelling technique will also allow strategic planning and optimization of public healthcare recruitment.

## Introduction

The lack of health workforce is a global crisis which numerous countries have proposed and implemented intervention plans [1, 2]. However, there is limited data regarding the impact of these interventions and their sustainability over a long period of time. Research shows that the loss of healthcare workers in African countries (such as South Africa and Ghana) cripples the pre-existing delicate health system [3, 4]. Hence, the retention of health workers is essential for the healthcare system performance. These studies also point out that the recruitment of health workers should not only focus on nurses and physicians, but also on community health workers (CHWs) to help the primary healthcare systems boost the coverage and address the basic health needs of societies [4].

Specifically, healthcare systems in sub-Saharan Africa (SSA) face a serious human resource crisis, with recent estimates pointing to a shortfall of more than half a million nurses and midwives needed to meet the Millennium Development Goals of improving the health and wellbeing of the SSA population by 2015 [5]. One of the reasons for this phenomenon is due to human capital flight (“brain drain”) in the health profession, especially in the public sector [1, 6]. Migration of health workers from low- and middle-income countries (LMICs) to high-income countries is a controversial aspect of globalization, having attracted considerable attention in health policy discourse at both the technical and political levels [1, 7–9]. The migration of skilled healthcare workforce translates into a direct loss of considerable resources to the public sector of LMICs, as direct benefits only accrue to countries, which have not invested in educating young professionals. To make matters worse, in many sub-Saharan countries such as Sierra Leone and South Africa, there are limited alternatives for the population to seek healthcare services from the private sector or next health facility due to inaccessible distance or cost factor [10].

To maintain a functional health system, most countries have altered their retirement age in order to extend the working life of their staffs. Furthermore, Botswana and South Africa have recruited from other countries within and outside the continent [7]. Despite various local and international frameworks, the effectiveness of these interventions is yet to be seen [7, 8]. Another challenge lies in the monitoring and evaluation of these frameworks. Recent cross-sectional reviews of currently available healthcare workforce database show that in most cases, the systems are fragmented, unreliable, and cannot be integrated at both national and international levels, and that in order for policy-makers to make data-driven decisions, better database management systems still need to be developed [1, 2, 8].

A high turnover rate in the health workforce is another concern as it is costly and detrimental to organizational performance and quality of care. Healthcare organizations with high attrition rate not only face issues with the quality, consistency and stability of services provided to people in need, but also issues regarding the working conditions of the remaining staffs such as increased workloads, disrupted team cohesion and decreased morale [11, 12].

Some studies have focused on the influence of individual and organizational factors on an employee’s intention to leave [13]. A World Health Organization (WHO) study of four African countries shows that the major reasons behind health worker migration are better salary, safer environment, living conditions, lack of facilities, lack of promotion, and heavy workloads [8]. Other studies conclude that better compensation package with good work-life balance is the primary reason to migrate [6, 14, 15]. On the other hand, one of the obstacles to migration is language barrier, which lies at the basis of patient care [16, 17]. Patients express their distress by describing their symptoms and pain and report changes in health status to professionals. Nurses or doctors need the current and technical language fluency to communicate under stress and duress with one another, members of the teams, and patient families [6].

Another healthcare policy concern is the misdistribution of healthcare workforce between urban and rural areas. It prevents equitable access to health services, contributes to increased health-care costs and underutilization of health professional skills in urban areas, and remains a barrier to universal health coverage [6].

Overall, the human capital flight of local health professionals, the high turnover rate, and the shortage of workers in the public sector of South Africa thus demands further investment in attracting and retaining foreign healthcare staffs that stay for an extended period of time. The WHO has also issued global recommendations to improve the rural recruitment and retention of the health workforce [18]. This is pivotal to the delivery of healthcare in rural and remote areas of South Africa. A study has shown that 84% of South African population uses public healthcare, served by only 30% of the trained and certified doctors [19]. Generally, sub-Saharan Africa faces severe lack of healthcare workers, with only 3% of the world’s total medical staff while facing 24% of the global burden of disease [8]. The arrival of foreign medical workforce and their placement in the public health sector reduces the two-front misdistribution of physicians, alleviates the lack of human resources in public rural facilities, and improves access to healthcare to people in rural areas [8].

To date, greater efforts have focused on recruitment, with significantly less attention to workforce retention. As aforementioned, a challenge to improve health access in rural areas is to maintain high retention rate of the medical workforce. Currently, there are few empirical studies regarding the factors that influence the length of practice [14, 17]. Previous attempts to identify these factors mainly focus on worker satisfaction at medical facilities and retention strategy of staffing agencies [17]. There are some recent research into the correlation between employee demographic information and the success of retention effort in public health facilities [14].

This paper aims to develop a predicting tool for the length of practice of foreign healthcare workers, given their demographic information. Machine learning methods are well-suited for this challenge. Rather than traditionally considering the effect of demographic variables on the length of practice one after another, machine learning method examines all potential predictors simultaneously in an unbiased manner, and identifies pattern of information that are useful to make prediction.

## Methods

### Study design

A quantitative retrospective cohort study was conducted using secondary data, collected from the Africa Health Placements (AHP).

### Study setting

South Africa Health, healthcare worker population in underserved communities and distribution and retention levels. AHP recruits foreign and locally qualified health professionals to be placed in underserved communities in South Africa. Underserved areas like rural areas often face challenges in recruiting and retaining health workers, government has responded with programmes like compulsory community service and rural allowance to address this challenge.

### Data acquisition

Longitudinal individual health worker records are maintained at AHP. These health workers included professionals from South Africa and the rest of the world seeking employment in underserved facilities in South Africa. Data was collected using two methods (i) customized online portal completed by healthcare workers (HCW) and (ii) interviews by recruitment officers through email, Skype, and telephonic conversations. Data were captured onto a database and customer management system called Docwize. The online portal is available at the AHP website as a contact form. Once registered, the HCW receives login details to complete their application on Docwize. This system allows them to input personal and professional information, upload certificates, which would then be verified with the respective regulatory authorities, and be informed about the next steps until they secured a job offer. The HCW have an option of completing the application online or supplying the details to the recruitment officers who then update the system. It takes an average of 18 months to complete the recruitment process, 75% of the HCW were discouraged by the regulatory delays resulting in incomplete data. The length of stay was continuously monitored during their employment contract. Emails and telephonic contact are used to establish their last date of employment at a particular facility.

### Statistical analysis

#### Dataset description and manipulation

We took a complete cases approach, using only data from successfully recruited health workers without missing observations. The Africa Health Placements dataset contains 62 variables and 13 698 entries, in which there were 2079 successfully recruited practitioners. Among these 2079 professionals, some chose not to provide personal information such as marital status or gender. After data cleaning, there were 1838 entries with completed fields to meet the requirements of this study.

The variables that are used to develop our machine learning models are chosen based on their availability in the AHP data system. They are nationality, profession, relationship, and gender. Since there are a lot of missing values in our age variable dataset, a complete case approach with age could have further reduced the dataset to merely 914 entries and undermine the ability of the model to learn from existing data. Hence, we excluded it from the final analysis. Notably, all of our four predictors are categorical variables. A challenge with having categorical variables in machine learning is that to fully represent each variable, we have to use a large number of dummy variables to represent each level within the variable. For example, since our data had records from 145 countries, we needed 144 dummy variables to represent all existing countries. This method would result in a very sparse dataset and usually not useful in predictive modelling. Hence, we transcribed each variable as follows:

- *Nationality*: categorical data of 145 different countries. Instead of recording nationality as it is, the nationality variable is transcribed based on World Bank’s classification of countries into 4 categories: low income, lower middle income, upper middle income, and high income.
- *Professions*: categorical data of 22 different registered professions, recorded into 3 different categories: doctor, nurse, and other
- *Gender*: categorical data of 2 levels: male and female
- *Relationship status*: categorical data of 3 levels: married, single, or other.

#### Machine learning model development

With a large recruitment and retention dataset from AHP, we built three machine learning predictive models using relevant demographic data. We evaluated the models’ performance by doing 10-fold cross-validation. The aim was to choose a model that performs significantly better in predicting length of practice.

As shown on Table 1, three different machine learning classification models (multinomial logistic regression, decision tree, and Naive Bayes Classification) were used to train the dataset. The issue was approached as a classification, rather than a regression problem, as we aimed to classify a successful recruit into one of the four mutually exclusive groups (less than 1 year, less than 2 years, less than 3 years, and more than 3 years). The use of a regression method is not optimal in this case, due to (i) the lack of quantitative numerical variables in our demographic information, (ii) the wide range of value of the dependent variables (length of practice measured in days), and (iii) the non-continuous nature of the dependent variables. A regression method would require a much larger dataset to arrive at a model of relatively acceptable fit. With our current available dataset, the experimental fit is approximately 18% with high internal sum of squares. Moreover, in strategic workforce planning, a precise prediction of the length of practice in days (or months) is generally not expected. A prediction of whether a specific healthcare worker will stay for 1 year, 2 years, or longer is usually acceptable for most intents and purposes.

**Table 1:** Machine learning results

	Techniques		
	Multinomial logistic	Decision tree	Naive Bayes
Accuracy	47.34% [1.63]	45.82% [1.69]	47.01% [1.62]
95% CI	(46.22, 50.84)	(46.66, 51.28)	(45.19, 49.81)
AUC	0.6652	0.6635	0.6602
No information rate [NIR]	0.376	0.376	0.376
*P* value [Acc > NIR]	< 2.2e−16	< 2.2e−16	< 2.2e−16
Cohen’s Kappa	0.2658	0.2649	0.2521

#### Cross-validation

To decide which of the three models perform best, we have to see their ability to generalize and predict new, unseen data. A challenge to our research was the lack of test data which we could have used for model evaluation. Conventionally splitting our existing data into a 80/20 ratio—80% of the data for training and 20% for testing—was an option, but not optimal as we wanted to use all data available for training.

We examined our three models with a technique called 10-fold cross-validation. Ten-fold cross-validation works as follows: we randomly partition the original dataset into 10 disjoint subsets, use nine of those subsets in the training process, make predictions about the remaining subset, and record the misclassification error. To avoid opportune data splits, we average misclassification error across the 10 folds. A comparison between the average misclassification errors of the three machine learning models allowed us to decide which model performs best on unseen data.

## Results

Three machine learning models were trained, and a 10-fold cross validation technique was used to attain evaluative statistics. The three models attain almost identical results, with negligible difference in accuracy. The “best”-performing model (multinomial logistic classifier) achieves a 47.34% [SD 1.63] while the decision tree model achieves an almost comparable 45.82% [SD 1.69] (Table 1).

Multiclass area under the curve (AUC) was computed by building multiple receiver operating characteristic (ROC) curves (one class versus another) and taking the average, as defined by Hand and Till [20]. The three models achieve an average AUC of 0.66 (multinomial logistic at 0.6652, decision tree 0.6635, Naive Bayes 0.6602), suggesting sufficient predictive signal at the four selected categorical variables.

Overall, the three models had significant accuracy in classifying the length of stay of healthcare workers (*p* value < 2.2e−16) (Table 1). Additionally, Kappa statistics was also computed, in order to measure how much better each of the classifiers is performing over the performance of a classifier that simply guesses at random according to the frequency of each class [21]. The Cohen’s Kappa statistics of the multinomial logistics, decision tree, and Naive Bayes are 0.2658, 0.2649, and 0.2521 respectively, suggesting a fair (but not substantial) agreement between prediction and response adjusted by the amount of agreement expected by chance.

All three models perform reasonably well at identifying those who are likely to stay for less than 1 year (Table 2). The sensitivity of this class was greater than 75% for all three models, showing that they correctly identify more than ¾ of those who are likely to stay less than 1 year. Specificity of this class is not particularly high (all lower than 65%), so all three models do not do as well in identifying those who are staying for more than 1 year. However, with a negative positive rate as high as 84% across the three techniques, it means that when the model negatively classifies a person out of those who stay for less than 1 year, such classification is likely to be correct.

**Table 2:** Predictions of length of stay across the three models

	Less than 1 year	Less than 2 years	Less than 3 years	More than 3 years
Multinomial logistic techniques				
Sensitivity	0.7685	0.3248	0.0369	0.5425
Specificity	0.6548	0.8503	0.9766	0.7896
Positive predictive value	0.5728	0.4533	0.2340	0.3700
Negative predictive value	0.8244	0.7673	0.8398	0.8834
Balanced accuracy	0.7166	0.5876	0.5068	0.6661
Decision tree techniques				
Sensitivity	0.7858	0.3740	0.000	0.4897
Specificity	0.6469	0.8075	1.000	0.8150
Positive predictive value	0.5728	0.4260	NaN	0.3761
Negative predictive value	0.8337	0.7716	0.8379	0.8751
Balanced accuracy	0.7164	0.5908	0.5000	0.6524
Naive Bayes techniques				
Sensitivity	0.7728	0.2658	0.0403	0.5630
Specificity	0.6391	0.8752	0.9760	0.7675
Positive predictive value	0.5633	0.4485	0.2449	0.3556
Negative predictive value	0.8236	0.7573	0.8401	0.8852
Balanced accuracy	0.7059	0.5704	0.5081	0.6653

**Fig. 1:**
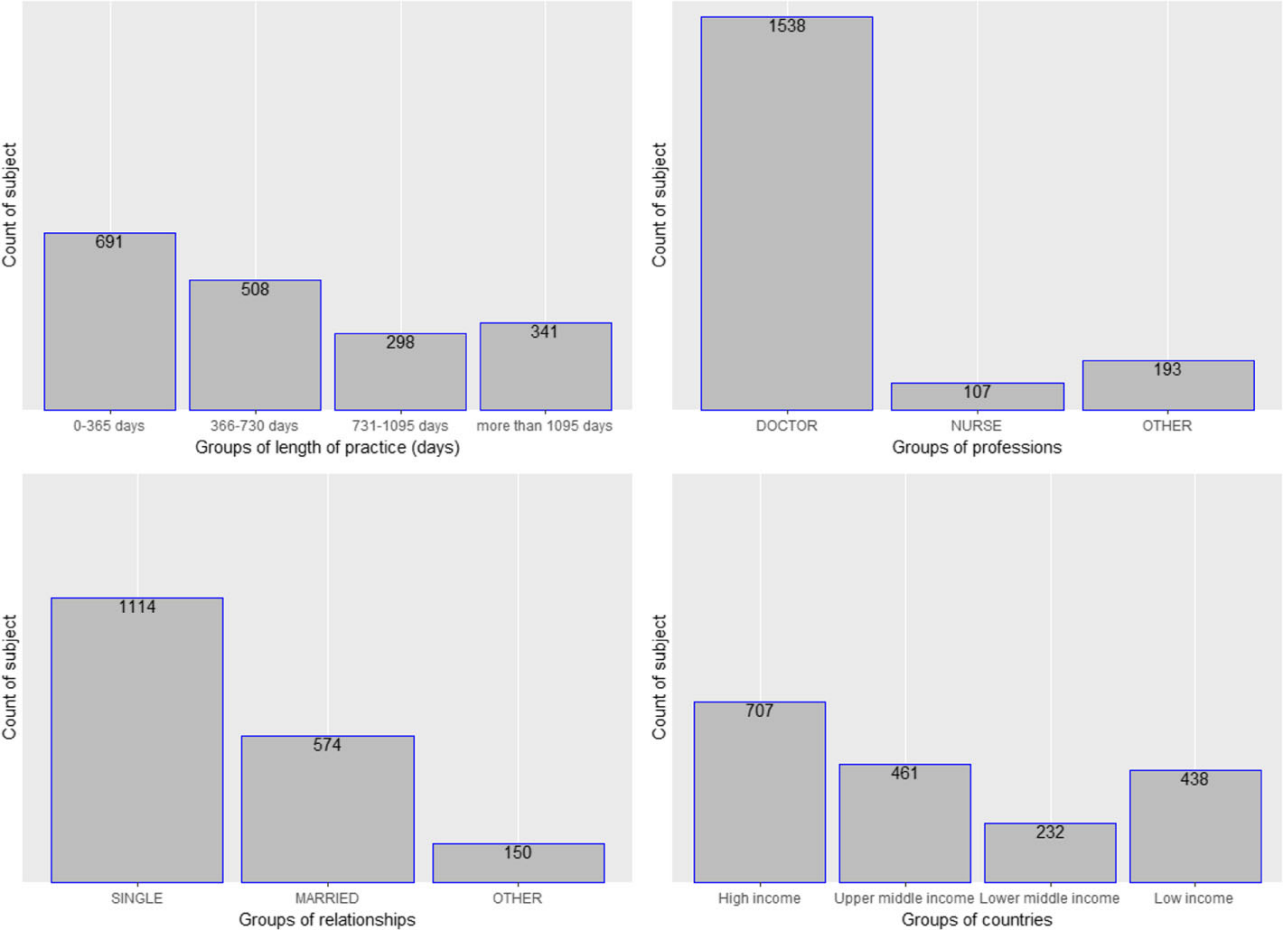
Number of subjects categorized by (from left to right, up to down) length of practice, professions, relationships, and countries

In contrast, all three models perform poorly at identifying those who are staying between 2 and 3 years (Table 2). With sensitivity at as low as 0% (decision tree) and specificity up to 100%, the three models must have learned to negatively assign a majority (all in decision tree case) out of this class. This is likely the result of imbalanced data sample with too little sample data of this class (Fig. 1).

### Comprehensive data analysis

**Fig. 2:**
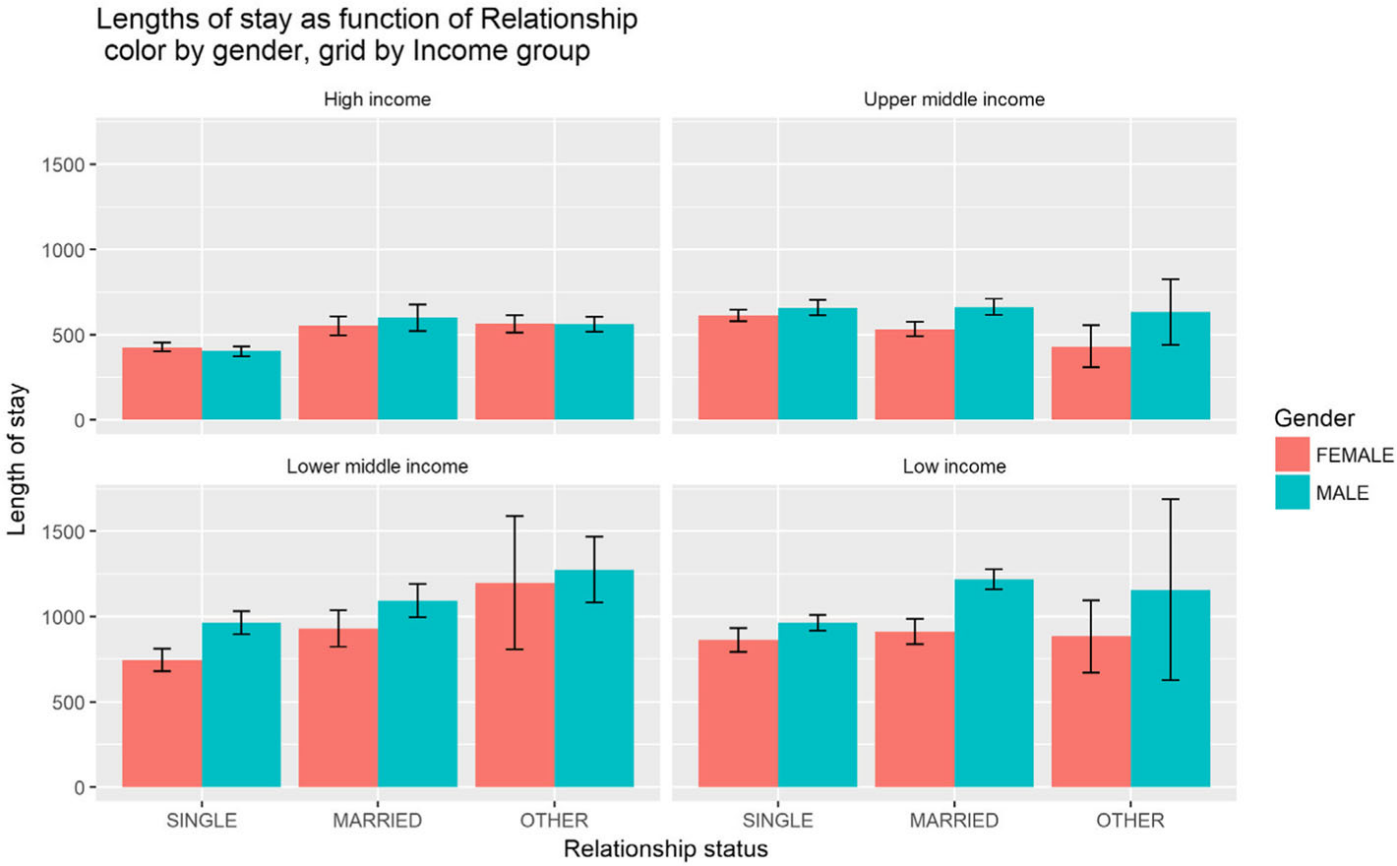
Length of stay as function of relationship, colour by gender and grid by income group

**Fig. 3:**
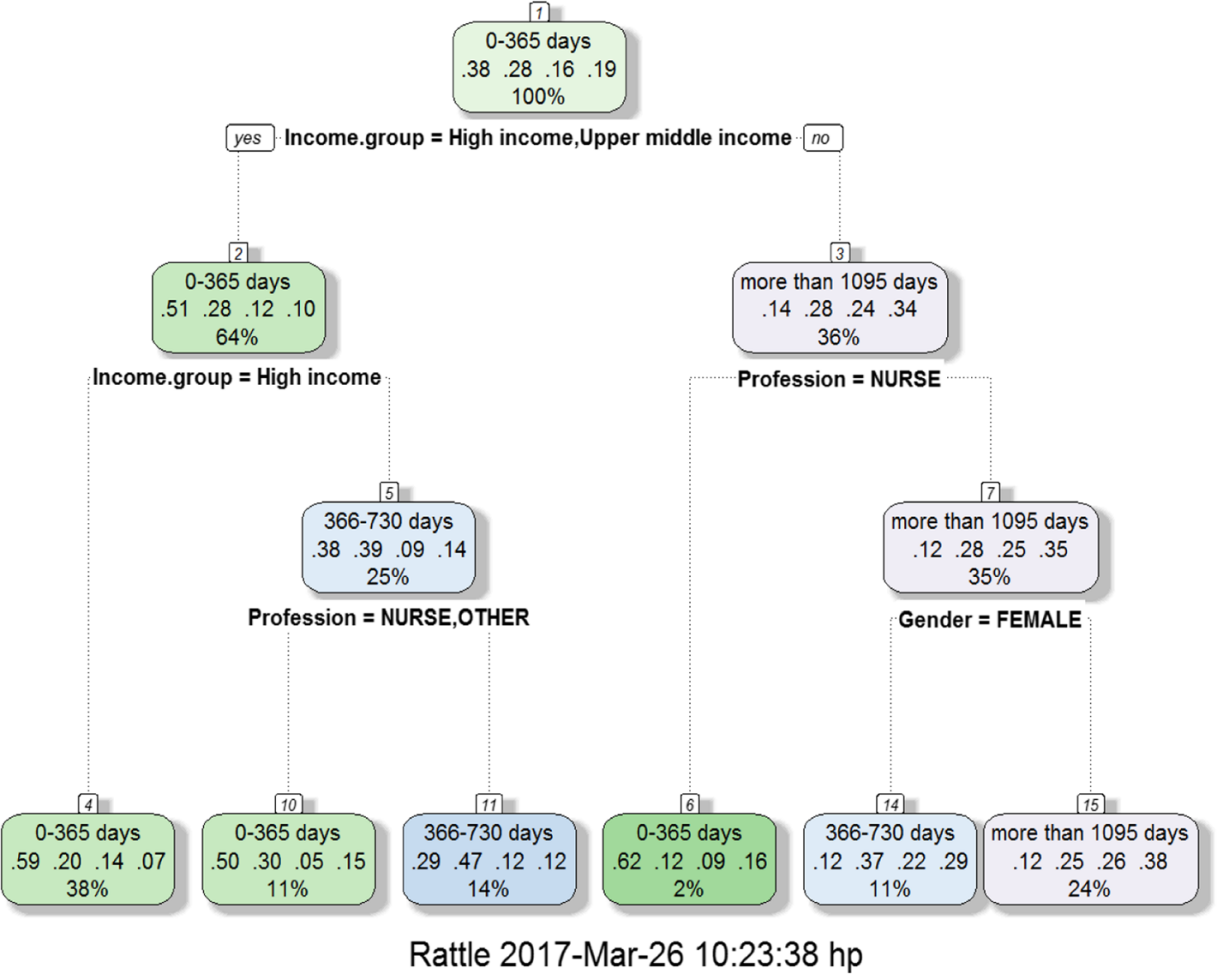
Decision tree on income, gender and profession

In general, more males (997, 54%) than females (861, 46%) were recruited (Table 3). Males stay on average 187.78 days more than females do. South Africa has supplied the greatest number of health workers (381, 41%), followed by the United Kingdom (361, 39%), Nigeria (106, 11%), and Netherlands (86, 9%) (Table 3). Doctors (1538, 83%) were the most recruited health workers and then nurses (107, 6%) and other professionals (193, 10%). With regard to relationship status, single healthcare workers constituted 61% of the recruited, 31% were married, and 8% were cohabiting (Table 3, Figs. 1, 2, and 3).

**Table 3:** Length of stay by gender, nationality, profession, and relationship status

	Mean length of stay (days)	Standard deviation (sd)	Sample (*n*)	Percentage (%)
Gender				
Female	603.48	499.0	861	46
Male	791.26	630.9	997	54
Total			1 838	100
Nationality (top 4)				
South Africa	548.65	388.1	381	41
United Kingdom	475.11	373.3	361	39
Nigeria	1 096.09	719.7	106	11
Netherlands	753.36	532.7	86	9
Registered profession				
Doctor	714.58	588.4	1 538	83
Nurse	575.38	498.2	107	6
Other supporting staff	684.31	550.9	193	10
Total			1 838	100
Relationship status				
Single	625.22	530.64	1 114	61
Married	868.46	659.26	574	31
Other	651.12	651.12	150	8
Total			1 838	100

**Fig. 4:**
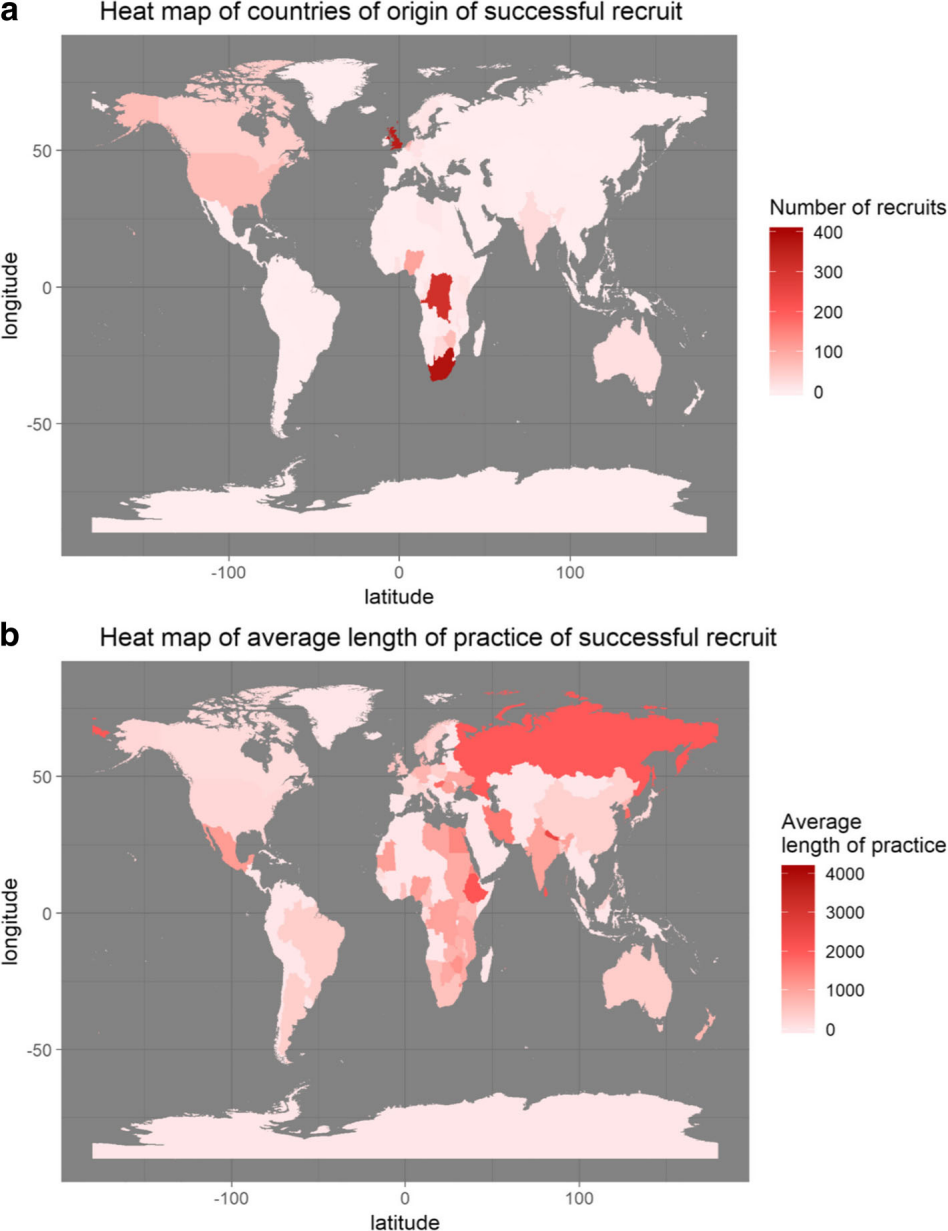
Map showing world distribution of **a** number of candidates sourced from each country and **b** average length of practice by these candidates from each respective country

Figure 4 shows two world heat maps that represent (a) the number of successful recruits from each country and (b) the average length of practice among those in these countries. The two maps point to an observation: AHP as a health placement organization is not very successful in recruiting from some countries, e.g. Russia, but once we do, the recruits tend to stay for an extended period of time. However, the sample size casts some doubts on this observation. Some countries have very high average length of stay, simply because we have a very small sample size of them.

## Discussion

This research shows that a majority of foreign qualified healthcare workers (1497 out of 1838, 81%) stay at their placement facilities for less than 3 years. While a constant rate of foreign recruitment per year can “fill the gap” in paper, the low average length of practice signifies a hidden cost of recruiting, relocating, and training of new healthcare professionals. Effective workforce planning from government or non-profit organizations, thus, requires a tool to predict the length of practice of incoming health professionals.

The three models attain significantly above chance results, with the average AUC of approximately 0.66 (multinomial logistic at 0.6652, decision tree at 0.6635, Naive Bayes at 0.6602), suggesting sufficient predictive signal at the four categorical variables selected. This is an indication that applying and retraining machine learning models with available datasets, Human Resource for Health decision makers can effectively source healthcare workers who are most likely to stay the longest in underserved communities.

Machine learning must be applied together with other qualitative methods like exit interviews so as to give an in-depth understanding of the healthcare worker perceptions and experiences that relate to their length of stay. A mixed method would have generated a better understanding of why certain gender, countries, age, and experience tend to stay longer than others.

### Limitations of the study

Incomplete fields in the data were another issue as many candidates were excluded from the study due to missing information. We could not obtain age as one of the predictors, although we recognized that it could potentially influence health worker long-term plan to stay. Our issue with incomplete data relates directly to the ineffective database system issue that is common among the public sector in South Africa [1, 2, 8]. Although in the short run, installing and enabling a more effective database system imposes a cost challenge to healthcare non-profits and public sector, such system is likely to make tremendous impacts as the machine learning models can be further improved by learning from a larger, high-quality dataset. In the meantime, there is a potential for the public sectors and NGOs to collaborate and involve in data sharing that could empower the training process of machine learning algorithms.

## Conclusions

Machine learning models give us an effective tool to predict the recruited health workers’ length of practice. These models can be adapted beyond the scope of demographic information (i.e. information about placement location, income), allowing strategic planning and optimization of public healthcare recruitment.

## Abbreviations

AUC: Area under the curve
HCW: Healthcare workers
LMIC: Low- and middle-income countries
NGO: Non-governmental organization
ROC: Receiver operating characteristic
SSA: Sub-Saharan Africa
WHO: World Health Organization
